# Serratia marcescens Canaliculitis: A Case Report of Uncommon Etiology

**DOI:** 10.7759/cureus.77259

**Published:** 2025-01-10

**Authors:** Josemaria M Castro, Armida L Suller-Pansacola

**Affiliations:** 1 Ophthalmology, Manila Doctors Hospital, Manila, PHL; 2 Ophthalmology and Visual Sciences, University of the Philippines-Philippine General Hospital (UP-PGH), Manila, PHL

**Keywords:** canalicular stones, canaliculitis, dacryolith, lacrimal canaliculitis, serratia

## Abstract

A 66-year-old female patient presented with chronic medial eyelid swelling and discharge of the right eye. She was initially treated with tobramycin eye drops and oral amoxicillin-clavulanic acid. However, the persistence of signs and symptoms accompanied by grainy punctal discharge on follow-up prompted a diagnosis of lacrimal canaliculitis. She underwent punctum-sparing right superior and inferior canaliculotomy under local anesthesia and completed topical antibiotic treatment with moxifloxacin. Serial follow-up examinations showed complete resolution of swelling, discharge, and epiphora. Microscopic examination of the lacrimal concretions showed the presence of *Serratia marcescens*, an unusual cause of canaliculitis. As a microorganism of nosocomial and opportunistic origin, it is commonly found in medical equipment, urine-collecting basins, and even tap water. Although extremely rare, *Serratia marcescens *should be considered in chronic lacrimal canaliculitis presenting with concretions.

## Introduction

The nasolacrimal system is essential in draining tears from the eyes into the nasal cavity through small channels called canaliculi and the nasolacrimal duct. Its intricate anatomy and variations make it prone to infections [1]. Canaliculitis is an acute or chronic infection of the canaliculi, which may present with epiphora, punctal edema, redness, and discharge. This condition is often misdiagnosed because it can mimic more common eye infections leading to prolonged discomfort and improper treatment. The most common cause of canaliculitis among bacterial anaerobes is *Actinomyces israelii* [2]. Other potential pathogens include *Streptococcus*, *Staphylococcus*, and fungal organisms [2]. We report an unusual case of lacrimal canaliculitis caused by *Serratia marcescens* in an otherwise healthy adult female patient. Related literature has also been included to supplement this case report. 

## Case presentation

**Figure 1:**
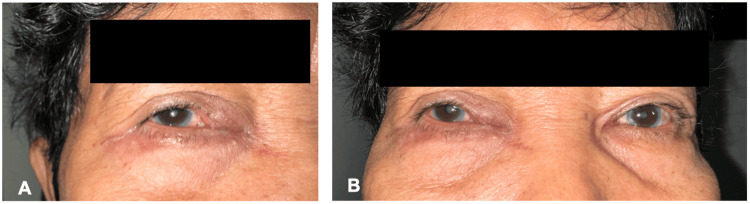
(A) Photograph on the patient's right periocular region showing edema and erythema of the medial aspect of the right upper and lower eyelids. (B) Comparison of the right and left periocular regions, with absence of inflammatory signs on the left side.

A 66-year-old female patient presented with a six-month history of tearing and progressively increasing mucoid discharge of the right eye. This was accompanied by tenderness and swelling of the medial aspect of her right upper and lower eyelids. Her medical, personal, social, and ophthalmic history were unremarkable. On initial examination, her best corrected visual acuity was 20/30 on the right eye and 20/20 on the left. Swelling, erythema, and tenderness of the canalicular portion of the right upper and lower eyelids and white mucoid punctal discharge were noted. The fluorescein dye disappearance test was negative on the right eye with Zappia and Milder grading 3 and positive on the left eye. The rest of the anterior segment and fundus examination findings were unremarkable. Lacrimal probing was deferred on the initial examination due to inflammation. Her clinical presentation was consistent with lacrimal canaliculitis, and she was started on tobramycin eye drops, oral amoxicillin-clavulanic acid, and oral celecoxib for seven days. There was improvement in the patient's symptoms on follow-up. However, there was persistent swelling of the right canalicular region, although reduced, as well as an associated grainy punctal discharge (Figure 1A, 1B). She was then advised to undergo lacrimal drainage surgery.

**Figure 2:**
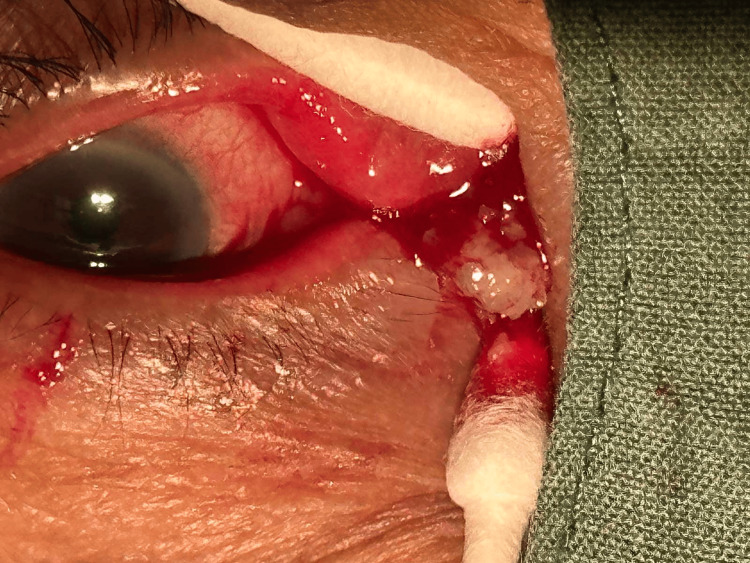
Photograph of the operative field showing canalicular concretions expressed during curettage.

The patient underwent punctum-sparing canaliculotomy, upper and lower, right side, under local anesthesia. The punctum was dilated with a lacrimal dilator and a Bowman probe was inserted. A "soft stop" was noted on both the upper and lower canaliculi. Intraoperatively, resistance was noted on probing at 4 mm in the upper canaliculus and 7 mm in the inferior canaliculus. A horizontal incision was made along the canaliculus 1 mm from the punctum followed by curettage of concretions (Figure 2). 

**Figure 3:**
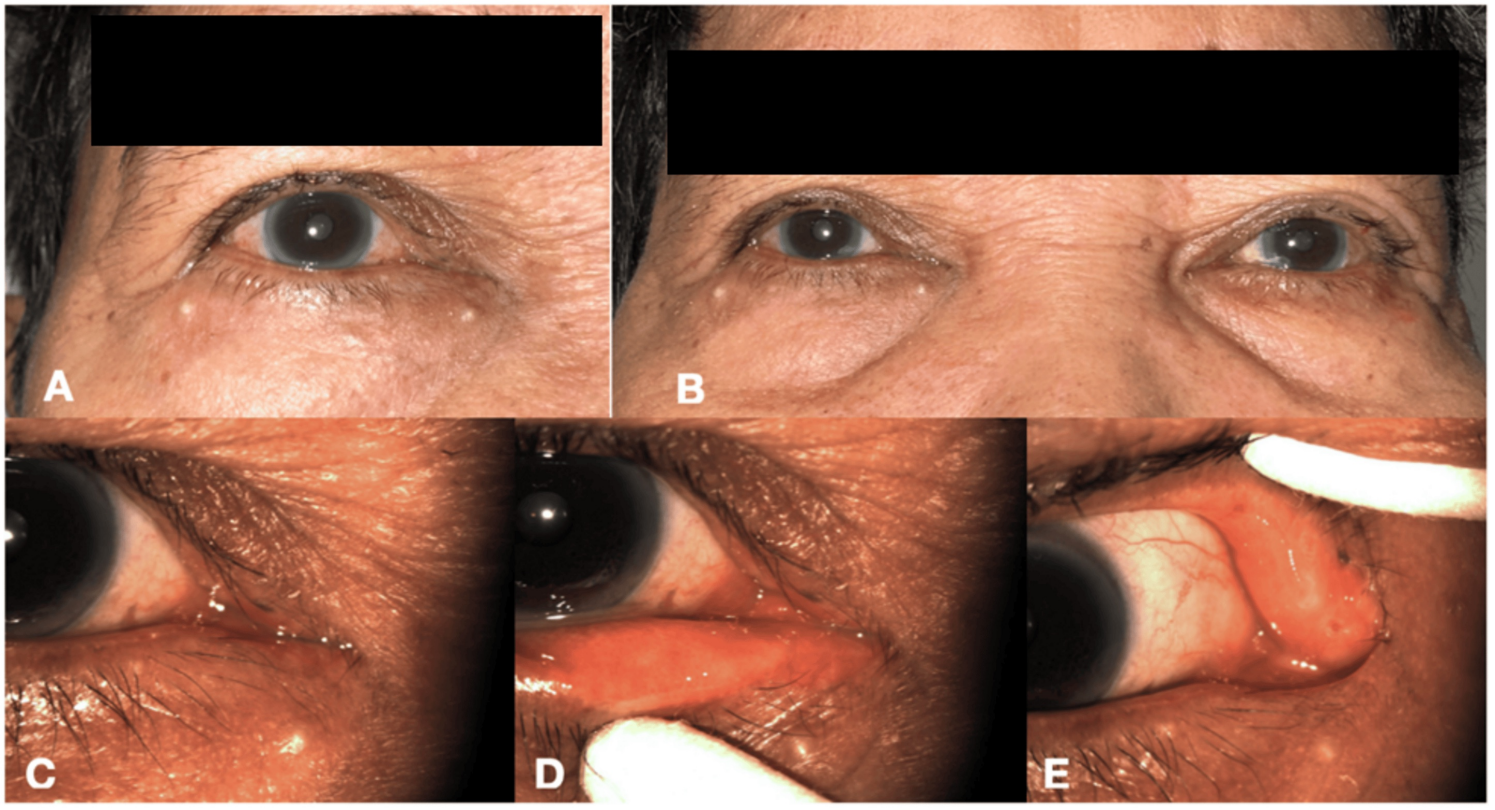
(A) Photograph at one month postoperatively showing complete resolution of eyelid swelling, edema, and discharge. (B) Comparison of right and left periocular areas. (C) Closer view of the canalicular region showing good apposition of eyelids to the globe. (D) Eversion of the right lower eyelid showing absence of punctal swelling, hyperemia, and discharge. (E) Eversion of the right upper eyelid showing absence of punctal swelling, hyperemia, and discharge.

The concretions or canalicular stones were sent for microbiological investigation. After removing the concretions, lacrimal apparatus irrigation with moxifloxacin was performed. The patient was then discharged and advised to continue moxifloxacin eye drops postoperatively for two weeks. No oral antibiotics were given. A serial examination was done on day 1, week 1, and month 1 post-surgery. There was a complete resolution of symptoms on the last day of follow-up, with a positive fluorescein dye disappearance test and Zappia and Milder grading 1 (Figure 3A-3E).

**Figure 4:**
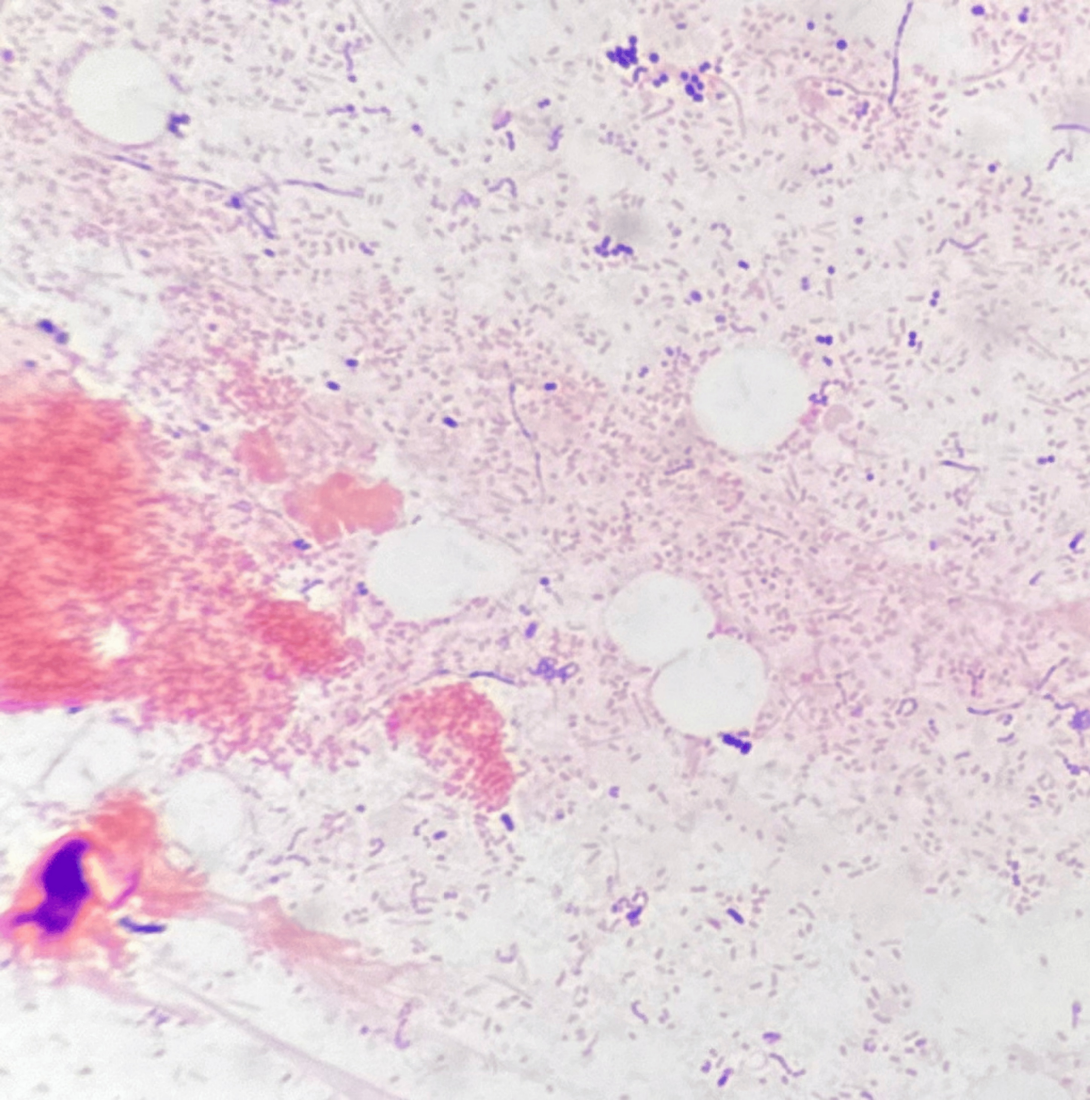
Microscopy of the canalicular concretions showing numerous Gram-negative bacilli (Gram stain with retention of Safranin dye, oil immersion, ×1000).

Microbiological studies of the canalicular concretions revealed exudative material with numerous Gram-negative bacilli (Figure 4). The culture yielded moderate growth of *Serratia marcescens* which was sensitive to the following antibiotics: piperacillin-tazobactam, cefotaxime, ceftriaxone, cefepime, gentamicin, ciprofloxacin, and trimethoprim/sulfamethoxazole. The microorganism was resistant to the following: amoxicillin, ampicillin, amoxicillin-clavulanic acid, ampicillin-sulbactam, cefaclor, cefalexin, cefalotin, cefazolin, cefonicid, cefamandole, cefuroxime, cefotetan, and cefoxitin. The patient was advised to complete the moxifloxacin eye drops, and no new antibiotics were given.

## Discussion

Canaliculitis is commonly misdiagnosed as conjunctivitis, preseptal cellulitis, and dacryocystitis. Primary canaliculitis may develop with no underlying cause and is usually due to *Actinomyces* or *Staphylococcus* infection [3]. It accounts for only 1-2% of all lacrimal diseases [4-6]. Patients with canaliculitis often complain of excessive tearing with discharge due to the blockage of canaliculi from severe inflammation and concretions.

Canaliculitis can be classified as either primary or secondary. Primary canaliculitis is an inflammation or infection that originates within the canaliculus, often caused by bacteria. Secondary canaliculitis occurs because of an extension of infection or inflammation from adjacent structures like in cases of conjunctivitis or dacryocystitis. Primary and secondary canaliculitis may have similar clinical presentations; however, the associated signs and symptoms can be influenced by the primary site of infection. In a major review of canaliculitis, it was found that the primary type was more frequent than the secondary, with the inferior canaliculus involved more commonly than the superior [7]. It also showed that *Serratia* comprised 0.59% (six out of 1009) of primary canaliculitis cases [7].

This case reports *Serratia marcescens* as an uncommon etiology of chronic canaliculitis in Asia and the first reported case in the Philippines. As members of the *Enterobacteriaceae* family, *Serratia* spp. are motile, non-endospore-forming Gram-negative rods [8]. They are opportunistic organisms of the respiratory and genitourinary system and have been reported to be nosocomial healthcare-associated pathogens [8]. As such, they are implicated in a wide range of infections including pneumonia, urinary tract infections, sepsis, meningitis, and endocarditis [8]. *Serratia marcescens* has been attributed to different sources like medical equipment and environmental sources such as air conditioning units, urine-collecting basins, bed-pan macerators, liquid soap dispensers, and even tap water [8]. Although this reported case did not have any comorbidities or history of prolonged hospital stay, the authors assumed that the patient likely contracted the infection from the use of contaminated tap water. 

There have been previous reports of contact lens-associated keratitis and keratoconjunctivitis from *Serratia *spp. [9,10]. Similar to other studies, the majority of the cases were responsive to fluoroquinolones [3,4,6,11-13]. A case of canaliculitis from *Serratia marcescens *has been reported in an elderly woman in Nigeria [11], but none has been documented in the Philippine setting to date. This case in Nigeria is similar to the current case because both patients had unremarkable clinical histories. Medial eyelid swelling and eye discharge for both cases were non-resolving with the initial use of topical antibiotics. However, in contrast to our case, the patient in Nigeria responded well to the topical and oral antibiotics and did not need any surgical intervention.

A study in India found other uncommon etiology causing canaliculitis which include *Corynebacterium*, *Enterococcus*, *Nocardia*, *Mycobacterium*, *Klebsiella*, *Pseudomonas*, *Citrobacter*, *Acinetobacter*, *Proteus*, *Aeromonas*, *Neisseria*, and *Aspergillus* [2]. Uncommon microorganisms were also reported to cause canaliculitis in a similar study in China and were identified to be *Stenotrophomonas*, *Propionibacterium*, *Peptostreptococcus*, *Fusobacterium*, *Capnocytophaga*, and *Eikenella* [12]. An observational study conducted in Saudi Arabia reported two out of 101 cases (1.9%) of *Serratia*-associated canaliculitis, along with other uncommon microbes such as *Gemella*, *Veillonella*, *Aerococcus*, and *Lactobacillus* [13].

*Serratia* has also been reported to cause nasolacrimal duct obstruction by the formation of dacryoliths [14]. In this study, *Serratia *was isolated in 6% (three out of 48) of cases with dacryoliths from primary canalicular obstructions and in 14% (one out of seven) of dacryoliths during dacryocystorhinostomy [14]. Consequently, other infections like canaliculitis may be a result of this chronic obstruction from dacryoliths [15].

Since canaliculitis can be caused by a wide range of microorganisms, culture and antibiotic sensitivity testing of the canalicular discharge and concretions are important to ensure appropriate antibiotic coverage. Inadequate treatment of canaliculitis leads to its recurrence and chronicity. Conservative management with topical antibiotics is often inadequate, and most patients will require surgery for the complete resolution of symptoms. Canaliculotomy with curettage of concretions is the gold standard in the treatment of canaliculitis [3,13,15]. This procedure is often performed under local anesthesia, with a small incision made from the punctum and continued along the length of the canaliculus. In some cases, a silicone stent may be placed to help keep the canaliculus open as it heals. Punctum-sparing canaliculotomy involves making the incision directly along the canaliculus without cutting through the punctum. This preserves the punctum's structure and function, allowing it to continue draining tears normally [16]. Punctum-sparing canaliculotomy with monocanalicular intubation has also been reported to be effective in a patient with upper and lower canaliculitis, which has a greater risk for epiphora compared to singly affected canaliculus [17].

## Conclusions

Canaliculitis may mimic more common eye infections and is often overlooked. A thorough history and ophthalmic examination including the canalicular region should always be done for patients presenting with epiphora. A high index of suspicion for canaliculitis is also important, especially for patients with recurrent and non-resolving eye redness, swelling, and discharge despite conservative management with topical antibiotics. *Serratia marcescens*, although rare, should be considered as a possible etiology in patients presenting with lacrimal canaliculitis.
